# Stress enhances hippocampal neuronal synchrony and alters ripple-spike interaction

**DOI:** 10.1016/j.ynstr.2021.100327

**Published:** 2021-04-13

**Authors:** Anupratap Tomar, Denis Polygalov, Sumantra Chattarji, Thomas J. McHugh

**Affiliations:** a Laboratory for Circuit & Behavioral Physiology, RIKEN Center for Brain Science, 2-1 Hirosawa, Wako-shi, Saitama, 351-0021, Japan; b National Centre for Biological Sciences, Bellary Road, Bangalore, 560065, India; c Centre for Discovery Brain Sciences, Deanery of Biomedical Sciences, University of Edinburgh, Hugh Robson Building, 15 George Square, Edinburgh, EH89XD, UK

**Keywords:** Stress, Hippocampus, CA1, SPW-Rs, Ripples, Pyramidal cells

## Abstract

Adverse effects of chronic stress include anxiety, depression, and memory deficits. Some of these stress-induced behavioural deficits are mediated by impaired hippocampal function. Much of our current understanding about how stress affects the hippocampus has been derived from post-mortem analyses of brain slices at fixed time points. Consequently, neural signatures of an ongoing stressful experiences in the intact brain of awake animals and their links to later hippocampal dysfunction remain poorly understood. Further, no information is available on the impact of stress on sharp-wave ripples (SPW-Rs), high frequency oscillation transients crucial for memory consolidation. Here, we used *in vivo* tetrode recordings to analyze the dynamic impact of 10 days of immobilization stress on neural activity in area CA1 of mice. While there was a net decrease in pyramidal cell activity in stressed animals, a greater fraction of CA1 spikes occurred specifically during sharp-wave ripples, resulting in an increase in neuronal synchrony. After repeated stress some of these alterations were visible during rest even in the absence of stress. These findings offer new insights into stress-induced changes in ripple-spike interactions and mechanisms through which chronic stress may interfere with subsequent information processing.

## 1 Introduction

The hippocampus is a medial temporal lobe structure that is crucial for encoding, updating and retrieving episodic memories (Eichenbaum, 2017; Tulving, 1985). Unfortunately, the same plasticity mechanisms that enable the hippocampus to perform these important functions also make it vulnerable to damage caused by severe and repeated stress (Chattarji et al., 2015; McEwen et al., 2015). Stress-induced changes in the rodent hippocampus include decrease in hippocampal volume (Schoenfeld et al., 2017) shrinkage and debranching of pyramidal cell dendrites, (Sousa et al., 2000), loss of dendritic spines (Magariños et al., 1997; Sandi et al., 2003), and alterations in synaptic plasticity mechanisms, including long-term potentiation (Alfarez et al., 2003; Shors et al., 1989). Together, these stress phenotypes at the cellular and synaptic levels are thought to contribute to impairments in hippocampus-dependent behaviour including learning and memory. However, our current understanding is based primarily on post-mortem analyses of stressed versus unstressed animals at fixed time points after the end of stress. The gradual and cumulative impact of stress on hippocampal function in the same animal over the course of repeated stress has not been explored in detail. Further, relatively little is known about how the intact, drug-free hippocampus is involved in the quick appraisal of an ongoing stressful situation (Cadle and Zoladz, 2015; Joëls, 2009).

These gaps in knowledge have been partially addressed by a handful of rodent studies that examined the effects of stress on *in vivo* hippocampal physiology during theta-associated foraging behaviour (Kim et al., 2007; Park et al., 2015; Passecker et al., 2011; Tomar et al., 2015), focusing on the activity of the hippocampal pyramidal (place) cells (O'Keefe and Dostrovsky, 1971) which form a cognitive map of the animals' surroundings (O'Keefe and Nadel, 1978). However, in addition to theta-associated exploratory states, pyramidal cells also exhibit highly coordinated activity during off-line behavioural states such as rest and sleep (Buzsáki, 1989). These offline states are dominated by high-frequency (100–200 Hz) transients termed sharp-wave ripples (SPW-Rs) which have been shown to play key roles in memory consolidation (Ego-Stengel and Wilson, 2010; Girardeau et al., 2009; Jadhav et al., 2012).

The altered learning and memory observed in stressed subjects (Kim et al., 2007; Park et al., 2015) raises the possibility that SPW-Rs characteristics and/or the ability of SPW-Rs to recruit CA1 cells (ripple-spike interactions) may be altered by stress. However, these important issues remain unexplored; the aim of the present study was to address them by analyzing hippocampal neural dynamics in mice on the first (acute) and last (chronic) day of a chronic immobilization stress (CIS) protocol. To characterize a neural signature of stress in area CA1 we employed high density tetrode recordings and assessed neuronal activity and local field potentials (LFPs) during stress (stress-state) and compared it with activity recorded in an adjacent quiescence/rest state (rest-state).

## 2 Material and methods

### 2.1 Animals

All experiments were performed using male C57BL/6J mice. In total 21 mice were used; of these, 4 were used for measuring the stress effects on bodyweight while the remaining 17 were used for *in vivo* electrophysiology. All mice were aged between 3 and 6 months at the start of experiments and were maintained on a 12-h light-dark cycle with *ad libitum* access to food and water. All procedures were approved by the RIKEN Institutional Animal Care and Use Committee and complied with the National Institutes of Health guide for the care and use of Laboratory animals (NIH Publications No. 8023, revised 1978). All efforts were made to minimize animal suffering and to reduce the number of animals used.

### 2.2 Experimental design and stress protocol

**Fig. 1:**
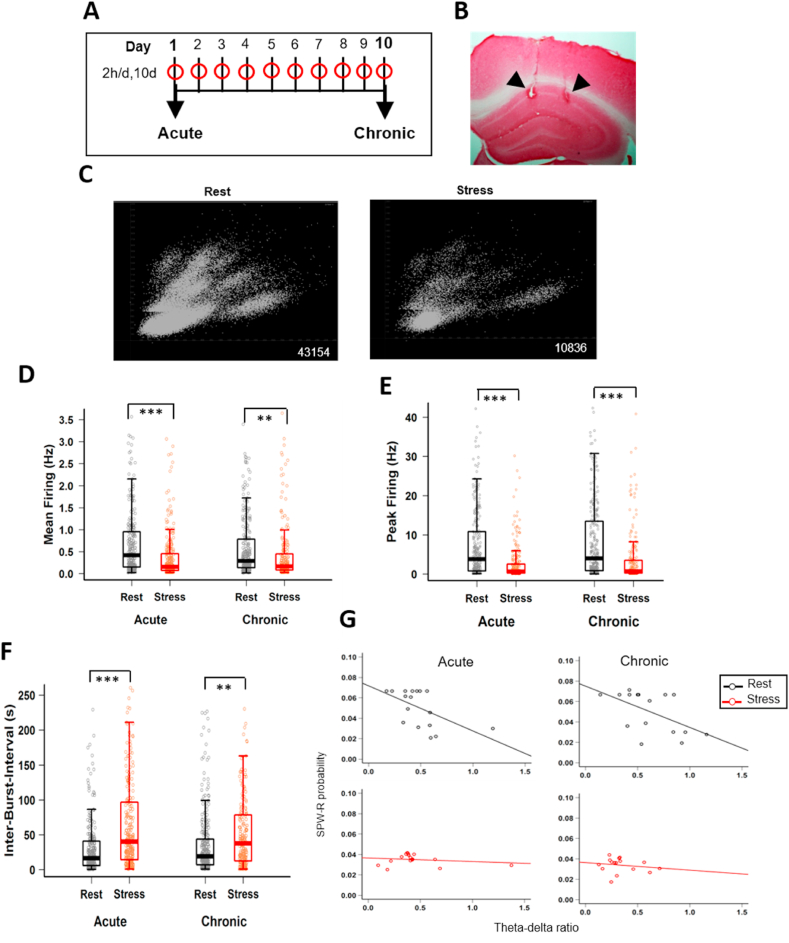
**CA1 pyramidal cell activity is altered during stress**. **(A)** Schematic representation of the chronic immobilization stress (CIS) protocol that mice received every day for 10 consecutive days. **(B)** Coronal section of the hippocampus showing the tetrode locations (black arrows) at the CA1 pyramidal layer. **(C)** Time matched (30 min) representative examples of unsorted spikes recorded during rest-state (left) and stress-state (right). Numbers next to each cluster depict total number of spikes recorded during that state. **(D)** Mean firing rate between rest-state and stress-state (two-way ANOVA: behaviour-state, F_(1, 1074)_ = 65.801, p = 1.35 × 10^−15^; day, F_(1, 1074)_ = 0.183, p = 0.669; behavior-state x day interaction, F_(1,1074)_ = 6.246, p = 0.013; Tukey's HSD: acute-rest vs acute-stress, p = 9.49 × 10^−13^; acute-rest vs chronic-rest, p = 0.095; chronic-rest vs chronic-stress, p = 0.0012). **(E)** Peak firing rate between rest-state and stress-state (two-way ANOVA: behavior-state, F_(1, 1074)_ = 142.510, p < 2.224 × 10^−16^; day, F_(1, 1074)_ = 2.971, p = 0.085; behaviour-state x day interaction, F_(1, 1074)_ = 0.817, p = 0.366; Tukey's HSD: acute-rest vs acute-stress, p = 6.41 × 10^−13^; chronic-rest, vs chronic-stress, p = 1.38 × 10^−12^). **(F)** Inter-burst-interval between rest-state and stress-state (two-way ANOVA: behaviour-state, F_(1, 1066)_ = 73.572, p < 2.22 × 10^−16^; day, F_(1, 1066)_ = 0.011, p = 0.916; behaviour-state x day interaction, F_(1, 1066)_ = 4.798, p = 0.029; Tukey's HSD: acute-rest vs acute-stress, 2.84 × 10^−13^; chronic-rest vs chronic-stress, p = 1.22 × 10^−4^). **(G)** Correlation between theta/delta ratio and SPW-Rs differs between behaviour-states on the first day (left: acute-rest, slope = −0.044, R = −0.56, p = 0.02; acute-stress, slope = 0.00, R = −0.19, p = 0.47) and last day (right: chronic-rest, slope = −0.04, R = −0.54, p = 0.029; chronic-stress, slope = −0.01, R = −0.17, p = 0.52) of CIS. All box plots represent interquartile range (IQR, 25th-75th percentiles), median is the thick line in the box and whiskers extend to 1.5 times the IQR. Circles depict rest-state (black) stress-state (red). *p < 0.05, **p < 0.01, ***p < 0.001. acute-rest: n = 288 cells, N = 17 mice, acute-stress: n = 282 cells, N = 16 mice, chronic-rest: n = 282 cells, N = 16 mice, chronic stress: n = 226 cells, N = 16 mice). (For interpretation of the references to colour in this figure legend, the reader is referred to the Web version of this article.)

Mice underwent the same chronic immobilization stress (CIS) protocol as described previously (Tomar et al., 2015). Briefly, mice experienced complete immobilization (2 h/d, 10 consecutive days: Fig. 1A.) in rodent immobilization bags, without access to food or water. All mice underwent the experimental protocol previously described, with the exception of 5 mice that also experienced a familiar track on the first and 10th day of experiment prior to stress exposure. Following surgery, mice were habituated to the small sleep-box on a daily basis during tetrode adjustments across 3–4 weeks; this is the same box in which all “rest” data was collected. Thus, this was already a highly familiar context and the mice were completely habituated to the experimenter, room, sleep box, etc., minimizing the contribution of other (non-stress) repetitive factors or experiences to the changes we observed in the physiology of the hippocampus between the two rest sessions. Here we examined the first 30 min of data recorded during CIS (stress-state) and the preceding quiescence period (rest-state). Recordings on the first day of CIS were termed ‘acute’ while those on the last day of experiment were termed ‘chronic’, providing four time points: i) acute-rest, ii) acute-stress, iii) chronic-rest and iv) chronic-stress. Rest-state data was recorded for ~15–30 min and hence for temporal distributions, theta/delta ratio, correlation between theta/delta and SPW-R occurrence, the first 15 min of data was used.

### 2.3 Surgery, recordings, and histology

Mice were anaesthetized using Avertin (2, 2, 2-tribromoethanol; Sigma-Aldrich, 476 mg/kg, i.p.) and were surgically implanted with a microdrive (manufactured with the assistance of the Advanced Manufacturing Support Team, RIKEN Center for Advanced Photonics, Japan). The microdrive housed eight independently movable tetrodes (14 μm diameter, nichrome) and was placed above right dorsal hippocampus (coordinates from bregma: AP -1.8 mm; ML + (1.2 mm). Prior to surgery, tetrodes were gold plated to lower impedance down to a range of 100–250 kΩ. Tetrodes were gradually lowered over the course of several days, such that by the start of the experiment they reached the CA1 stratum *pyramidale*. Data were acquired using a 32-channel DigitalLynx 4S acquisition system (Neuralynx, Bozeman, MT). Signals were sampled at 32,556 Hz and spike waveforms were filtered between 600 Hz and 6 kHz. Skull screws located above the cerebellum served as a ground, and a tetrode that was seated in the superficial layers of the neocortex, and devoid of spiking activity, was used for referencing. 3–4 weeks after surgery, when all tetrodes reached the CA1 stratum *pyramidale*, evident by multiple large amplitude spikes and SPW-Rs, the experiment was initiated. To ensure maximum unit yield and to avoid tetrode drift, which is usually in the downward direction, fine adjustments in tetrode positions were made between days which included either lowering or raising tetrodes.

During both rest-state and stress-state recordings the mice were located in a small circular sleep box (15 cm diameter). At the conclusion of the experiment mice underwent terminal anaesthesia (Avertin), and electric current (30 μA, for 8 s) was administered through each electrode to mark their locations. Transcardial perfusion was carried by using saline followed by 4% paraformaldehyde (PFA) followed by a further 24 h fixation in 4% PFA. Brains were sliced using a vibratome (Leica) to prepare coronal slices (50 μm thick), which were subsequently stained with Eosin Y, and inspected by standard light microscopy to confirm electrode placement.

### 2.4 Unit isolation and spike analysis

Spike sorting was performed by an automatic spike sorting program (KlustaKwik (Harris et al., 2000);), followed by manual adjustments of the cluster boundaries using SpikeSort3D software (Neuralynx). Candidate clusters with <0.5% of spikes displaying an inter-spike-interval shorter than 2 ms, a total number of spikes exceeding 50, having a cluster isolation distance value (Schmitzer-Torbert et al., 2005) >15, spike width (peak-to-trough) >170 μs and complex spike index (CSI) (McHugh et al., 1996) >5 were considered as pyramidal cells and were used for further analysis.

### 2.5 Local field potential analysis

The raw LFP data were first down-sampled using a custom software written in C to 1627.8 Hz (a factor of 20), followed by a quality control measure and channel selection via visual inspection and largest power in SPW-R frequency band (80–250 Hz). A low-pass filter with a cut-off frequency equal to half the target sampling frequency was applied to the LFP prior to down-sampling to prevent any signal distortion. Power spectral density (PSD) was calculated by using Welch's averaged modified periodogram method (*pwelch* function in Matlab) with a 2048 sample window size (1.26 s), 50% overlap and 4096 FFT points (2.52 s) resulting in a time-varying spectrogram. To account for power fluctuations caused by differences in position/impedance of the electrodes and to make PSD values comparable across mice, each PSD curve was normalized by its own mean power within the 0–3 Hz frequency band. Temporal dynamics between behaviour states were assessed by binning LFP data in 1 min time-bins and by comparing this data between stress-states and rest-states.

### 2.6 SPW-R detection

SPW-R events were detected using modifications to the method described in (Csicsvari et al., 1999a). As we did not have a clear hypothesis about the impact of stress on SPW-R frequency, we used a broad filter setting (80–250Hz) as described in Nakashiba et al. (2009) in order to have the parametric space to observe both increases and decreases in average frequency. Previously selected LFP channels were first band-pass filtered (80–250 Hz) using a 69 order Kaiser-window FIR zero-phase shift filter. Subsequently, the absolute value of Hilbert transform (instantaneous ripple power) was smoothed with a 50 ms Gaussian window and candidate SWR-events were detected as periods where the magnitude exceeded 3 standard deviation (SD) above its mean for longer than 30 ms. Further, the initiation and termination points of candidate SWR events were defined as points when the magnitude returned to the mean. Summed multi-unit activity (MUA) across all pyramidal cells that fired during any given recorded session was converted to instantaneous firing rate using time bin size equal to the underlying LFP trace sampling rate and smoothed, to allow detection of firing bursts using the same thresholds as described for the candidate SPW-R events detection. Candidate SPW-R events not coincident with MUA bursts were excluded from subsequent analysis. For precise peak PSD frequency detection of the SPW-R events, a multitaper method was employed on the product of each filtered SPW-R waveform and Hanning window of the same length.

### 2.7 Single unit properties

Mean firing rate of individual single units was calculated as the total number of spikes emitted by the unit during sleep/stress trial divided by the trial's duration. Peak firing rate of each single unit was calculated by smoothing ISIs of the unit with a 5SD Gaussian kernel and taking the maximum value of the resulting firing rate curve (firing rate over time). Spike bursting analysis was performed as described previously (Bakkum et al., 2014) by defining two or more spikes occurring within 10 ms time bin as a burst.

### 2.8 SPW-R triggered spectrograms

The spectrograms were calculated by using complex wavelet transform (CWT) (Morlet type wavelet, parameter = 7) method applied to a segment of the wide band LFP in 400 ms window centred on each SPW-R. Resulting spectrograms were averaged across individual SPW-R events in each recording session/trial. To compensate for 1/f power loss each power value within each spectrogram was multiplied with the frequency correspondent to that power value. To make power values comparable across subjects, each spectrogram was normalized by its own mean power within (0–5 Hz) frequency band.

### 2.9 SPW-R bursting analysis

The concentration of SPW-R events in time (bursting) was estimated by first taking the middle time stamp of each SPW-R event and calculating the difference between time stamps of the previous and next event. If adjacent events occurred further than 200 ms apart the current event was marked as ‘singlet’. The number of timestamps in remaining events was counted, and each event marked as a member of ‘doublet’, ‘triplet’ or ‘other’ (for more than 3 events in a SPW-R burst).

### 2.10 Participation of single units in SPW-R events

Each ripple event's start and end timestamps were used to quantify single unit activity corresponding to the co-occurred SPW-R events. Within SPW-R firing rate of any given single unit within each trial was calculated as the total number of spikes generated by the single unit divided by combined duration of all SPW-R events of the trial. Between SPW-R firing rate for any given trial was calculated by first, removing all spikes fired within all SPW-R events that occurred during the trial and then calculating the mean firing rate of resulting spike train. Participation of a single unit in SPW-R events was calculated as the percentage of SPW-R events the unit fired at least single spike in during any given trial.

### 2.11 Coactivity Z-score

A likelihood of any given pair of single units (limited to pyramidal cells) firing together during SPW-R events or “coactivity Z-score” was calculated as described previously (Singer and Frank, 2009). Briefly, for any given trial a set of start and stop timestamps of SPW-R events and a set of spike train timestamps, fired by pyramidal cells were prepared. Then a ‘coactivity matrix’ of size (N cells x N SPW-R events) was calculated. Each element of the coactivity matrix is set to 1 if a given cell was active (e.g., fired at least one spike) during given SPW-R event or 0 otherwise. Next, for every possible pair of cells a raw coactivity score was calculated as the number of SPW-R events during which both cells of the pair were active. Finally, a z-scored coactivity value was calculated by normalization of the difference between raw coactivity score and variance by standard deviation as described in (Singer and Frank, 2009).

### 2.12 Ripple-phase locking analysis

The phase relationship between SPW-R waveforms and spikes was calculated by first filtering whole LFP traces in the ripple band (80–250 Hz) using a two-way least-squares FIR filter. The filtered signal was then converted into phase values (+/- 180°) using the Hilbert transform. Peaks in the phase values (points corresponding to 180-degree angles) were detected and each spike of every spike train was assigned a phase value using the interpolation method which is not sensitive to the ripple wave asymmetry. Spikes fired outside of SPW-R events were removed and the remaining set of spike phase values was converted into firing probability histograms (10-degree bin size). Only cells significantly locked to SPW-Rs events were used in the analysis.

### 2.13 Statistical analysis

All statistical analyses were performed in R software (3.3.2). To test if data were normally distributed, the Shapiro-Wilk test of normality was used. All boxplots were analyzed using non-parametric statistics where data were first ranked and then two-way ANOVAs (*aov* function, stats package) followed by Tukey's honestly significant difference (HSD) tests were run on the ranks (*TukeyHSD* function, stats package). Similarly, distributions of various SPW-R properties as a function of SPW-R duration/amplitude were assessed by first ranking the data and then employing linear mixed effects models (LMMs) on this ranked data, where mouse identity was specified as a random factor and behaviour states and categorical variables were specified as fixed factors (*lmer* function, lme4 package). The output of the *lmer* function was summarized as an ANOVA table (*anova* function, stats package). Post hoc pair-wise comparisons were made using the least-squares-means (LSM) approach (*lsmeans* function, lsmeans package). Correlation between parameters was calculated using Pearson's correlation coefficient analysis (base package). Dependence of a parameter on another was calculated by employing standardized major axis (SMA) regression (*sma* function, smatr package). For the log-transformed scatter plots, the slope and the R^2^ values were calculated using log-transformed data and accordingly reported. Comparisons between regression lines were made by likelihood ratio tests (*sma* function, smatr package). For cumulative distribution analysis, the Kolmogorov-Smirnov test was employed (*ks.test,* stats package). Boxplots represent Interquartile Range (IQR, 25th-75th percentiles), median is the thick line housed in the box and whiskers extend to 1.5 times the IQR. No data point was removed as an outlier either for making boxplots or for statistical analysis. Unless noted, the level of statistical significance was set to 0.05 and p values are shown as follows: *p < 0.05; **p < 0.01; ***p < 0.001.

## 3 Results

This was designed as a longitudinal study, with recordings from the same cohort of mice during both rest and stress at acute and chronic time points providing samples from each mouse in a single group of animals across time and state. We employed a 10-day chronic immobilization stress (CIS) paradigm (Fig. 1A), that has been previously used to examine the effects of chronic stress on hippocampal memory, volume and CA1 spatial coding (Rahman et al., 2016; Tomar et al., 2015). Specifically, we compared hippocampal activity during the first episode of stress (stress-state) to the preceding stress-free period (rest-state) on Day 1; this is referred to as the “acute” condition as this involves only a single exposure to stress. In other words, the effects of acute stress on the first day of CIS were analyzed by comparing two conditions: i) acute-rest, and ii) acute-stress. Second, we carried out the same analyses on the last day of CIS when the same animal has already experienced 9 exposures to the same stressor. Thus, we again compared activity during the 10th episode of stress (stress-state) to an adjacent stress-free period (rest-state). This is referred to as the “chronic” condition as it involves quantifying the cumulative effects of repeated stress over 10 days. Here again we compared two more conditions: iii) chronic-rest, and iv) chronic-stress, and together data from these four conditions is presented in the following sections.

### 3.1 CA1 pyramidal cell spiking differs between stress-state and rest-state

The CIS protocol led to a gradual decrease in body weight (acute, 28.82 ± 0.97 vs chronic, 26.65 ± 0.69, N = 4 mice, paired *t*-test: t = 3.8411, p = 0.031) (Supplementary Fig. 1A), confirming the efficacy of this chronic stress paradigm, as it is consistent with previous reports (Vyas et al., 2002). Lesions in the stratum *pyramidale* confirmed that recordings were made from area CA1 (Fig. 1B.).

A long-held view on the detrimental effects of stress centers on the idea that severe and repeated stress leads to hippocampal hyperactivity, which in extreme cases may cause excitotoxic damage. For instance, both the glucocorticoid cascade (Sapolsky, 1996) and synaptic saturation hypotheses of stress (Cadle and Zoladz, 2015; Diamond et al., 2004) suggest that enhanced calcium and glutamate release during stress alter subsequent synaptic plasticity and mnemonic processes. The implicit assumption underlying both these hypotheses is that hippocampal neuronal networks undergo hyperexcitability during stressful experiences, though no study has directly tested this possibility in the intact brain *in vivo*. Surprisingly, we found hippocampal multiunit activity to be suppressed during stress (Fig. 1C and Supplementary Figs. 1B and C). Comparison of the average firing rate of CA1 pyramidal cells between stress and rest across days revealed a significant decrease during the stress-state (Fig. 1D) with the difference being most pronounced during acute stress on the first day (acute-rest, 0.80 ± 0.06 Hz vs acute-stress, 0.38 ± 0.03 Hz, p = 9.49 × 10^−13^, Tukey's HSD). A small but significant decrease in mean firing was noticed on the last day of stress (chronic-rest, 0.59 ± 0.04, vs chronic-stress, 0.48 ± 0.05, p = 0.0012, Tukey's HSD), with no significant difference between rest-states across days (acute-rest, vs chronic-rest, p = 0.095, Tukey's HSD). **S**imilarly, we observed a significant lowering of peak firing rates (Hz) (Fig. 1E**)** on both the first day (acute-rest, 7.77 ± 0.59 Hz, vs acute-stress, 3.37 ± 0.49 Hz, p = 6.41 × 10^−13^, Tukey's HSD) and the last day (chronic-rest, 8.80 ± 0.69 Hz vs chronic-stress, 4.31 ± 0.62 Hz, p = 1.38 × 10^−12^, Tukey's HSD) of CIS and no significant difference was noticed between rest-states across days (acute-rest, vs chronic-rest, p = 0.997, Tukey's HSD). While stress did not alter the length (ms) of bursting activity, the stress-state was associated with longer inter-burst-intervals (IBI) (Fig. 1F), indicating that time gaps (s) between burst activity were lengthened, consistent with the overall decrease in activity (acute-rest, 33.29 ± 3.33 vs acute-stress, 78.69 ± 6.42, p = 2.84 × 10^−13^; chronic-rest, 40.41 ± 3.68 vs chronic-stress, 65.33 ± 6.82, p = 1.22 × 10^−4^), with no difference in IBI observed between rest-states across days (acute-rest, vs chronic-rest, p = 0.313, Tukey's HSD). Taken together, these data demonstrate that stress exposure decreases the overall activity of CA1 pyramidal cells.

### 3.2 LFP profile and SPW-R properties differ between the stress-state and rest-state

Considering stress did not cause an increase in CA1 firing rates, we next focused on LFP based measures of excitability (Buzsáki, 2015). Hippocampal LFP patterns differ across behavioural states (Buzsáki, 1989) and hippocampal excitability is elevated during offline states such as rest, immobility and sleep (Grosmark et al., 2012), which are defined by a low ratio of LFP power between the theta (6–12 Hz) and delta (1–4 Hz) bands, along with periodic high-frequency sharp-wave ripples (SPW-Rs, 120–200 Hz). These are unlike exploratory-states that display theta oscillations with minimal occurrence of delta oscillations and SPW-Rs or attentive immobile states, in which slower theta oscillations dominate (Kramis et al., 1975). Indeed, during the unstressed rest-state we observed the expected inverse correlation between the theta/delta ratio and SPW-R occurrence probability. However, this relationship was altered during both acute (Fig. 1G, left; acute-rest, R = −0.56, p = 0.02, vs acute-stress, R = −0.19, p = 0.47) and chronic stress (Fig. 1G, right; chronic-rest, R = −0.54, p = 0.029 vs chronic-stress, R = −0.17, p = 0.52). The observation that stress-state is associated with a lower theta/delta ratio suggests the stress-state bears similarities to a rest-state, yet the absence of increased probability of ripple occurrence indicates that mice were likely not asleep. Thus, we consider the physiological state during chronic stress as similar, yet distinct from the typical rest-state.

**Fig. 2:**
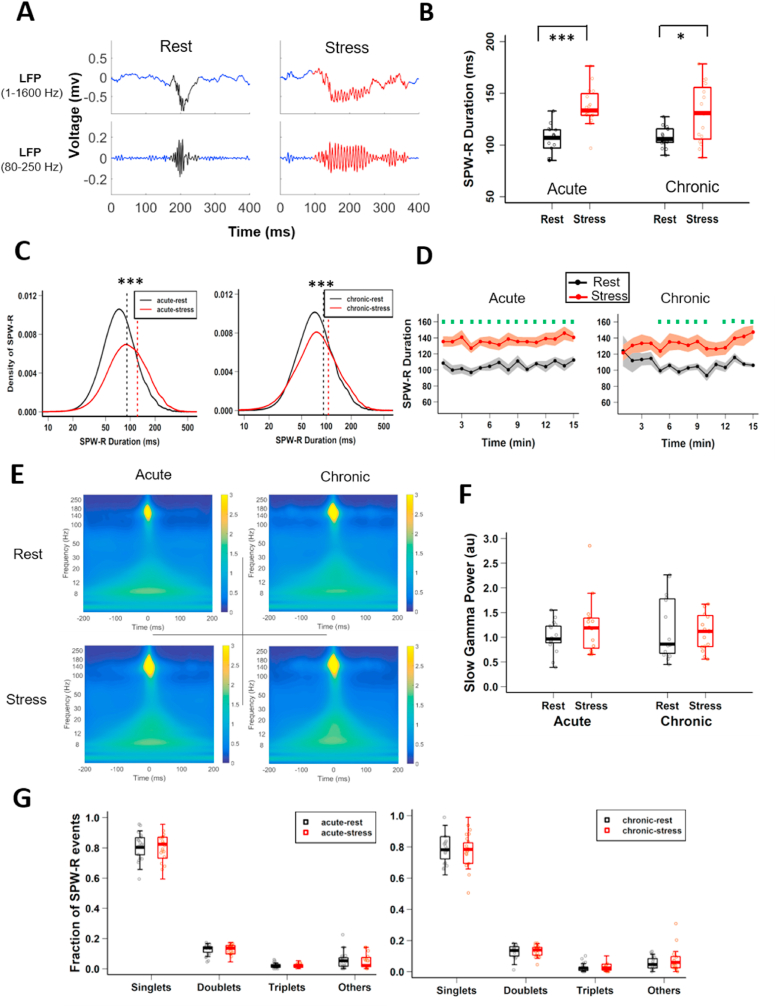
**CA1 SPW-R duration differs between rest and stress states. (A)** Representative examples of local field potential (LFP) for non-filtered (top) and filtered (bottom) SPW-R events recorded during rest-state (left) and stress-state (right) from CA1 *stratum pyramidale*. **(B)** SPW-R duration differs between rest-state and stress-state; (two-way ANOVA: behaviour-state, F_(1, 61)_ = 34.035, p = 2.22 × 10^−7^; day, F_(1, 61)_ = 0.419, p = 0.520; behaviour-state x day, F_(1, 61)_ = 1.813, p = 0.183; Tukey's HSD: acute-rest vs acute-stress, p = 2.07 × 10^−5^, chronic-rest vs chronic-stress, p = 0.0137). **(C)** Distributions of the duration of SPW-Rs differs between behaviour states on first day (left: acute-rest vs acute-stress, Kolmogorov-Smirnov test, D = 0.236, p < 2.22 × 10^−16^) and last day (right: chronic-rest vs chronic-stress, Kolmogorov-Smirnov test, D = 0.113, p < 2.22 × 10^−16^) showed a rightward shift. Dotted vertical lines represent median values. **(D)** Temporal dynamics of averaged SPW-R duration (1-min bins) differ between behaviour states on the first day (left: LMMs; behaviour-state, F_(1, 447)_ = 290. 40, p < 2.22 × 10^−16^; time, F_(14, 447)_ = 0.757, p = 0.716; behaviour-state x time, F_(14, 447)_ = 0.447, p = 0.958). Similarly, on the last day, SPW-R duration increased during stress (right: LMMs; behaviour-state, F_(1, 412)_ = 94.08, p < 2.22 × 10^−16^; time: F_(14, 412)_ = 1.19, p = 0.276; behaviour-state x time, F_(14, 412)_ = 0.631, p = 0.839). **(E)** Averaged peri-SPW-R wavelet spectrograms during acute-rest (top left), acute-stress (bottom left), chronic-rest (top right) and chronic-stress (bottom right). **(F)** Low gamma (17–40Hz) power during SPW-R events does not differ between behaviour-states (two-way ANOVA: behaviour-state, F_(1, 61)_ = 0.80, p = 0.38; day, F_(1, 61)_ = 0.008, p = 0.928; behaviour-state x day, F_(1, 61)_ = 0.105, p = 0.75). **(G)** Fraction of different types of ripple bursts during acute stress (two-way ANOVA: behaviour-state, F_(1, 124)_ = 0.374, p = 0.542; category, F_(3, 124)_ = 156.68, p < 2.22 × 10^−16^; behaviour-state x category, F_(3, 124)_ = 0.211, p = 0.89; Tukey's HSD: acute-rest vs acute-stress: singlets, p = 0.999; doublets, p = 0.998; triplets, p = 0.999; others, p = 0.996) and chronic stress (two-way ANOVA: behaviour-state, F_(1, 120)_ = 0.068, p = 0.795; category, F_(3, 120)_ = 147.2, p < 2.22 × 10^−16^; behaviour-state x category, F_(3, 124)_ = 0.371, p = 0.774; Tukey's HSD: chronic-rest vs chronic-stress: singlets, p = 1.0; doublets, p = 0.999; triplets, p = 0.967; others, p = 1.0). All box plots represent median and 25th-75th percentiles, with whiskers extending to the extreme data points. Circles depict rest-state (black) stress-state (red). *p < 0.05, **p < 0.01, ***p < 0.001. Acute-rest: n = 7220 SPW-Rs, N = 17 mice; acute-stress: n = 9851 SPW-Rs, N = 16 mice; chronic-rest: n = 7041 SPW-Rs, N = 16 mice; chronic stress: n = 8978 SPW-Rs, N = 16 mice). (For interpretation of the references to colour in this figure legend, the reader is referred to the Web version of this article.)

Growing evidence has linked altered SPW-R properties to hippocampus-dependent memory (Fernández-Ruiz et al., 2019; Nakashiba et al., 2009), a process affected by chronic stress (Kim et al., 2007; Park et al., 2015). Hence, we next analyzed SPW-R properties in both the early and late stages of chronic stress. While no differences were observed in the intrinsic oscillation frequency (Supplementary Fig. 2A), the average SPW-R duration was significantly longer during stress at both acute and chronic time points (Fig. 2B; acute-rest, 104.94 ± 3.29 ms vs acute-stress, 137.05 ± 4.65 ms, p = 2.07 × 10^−5^; chronic-rest, 107.54 ± 2.44 ms vs chronic-stress, 130.35 ± 6.996 ms, p = 0.0137, Tukey's HSD). The population distribution of SPW-R duration (Fig. 2C) clearly showed a greater fraction of long-duration ripples during both acute and chronic stress (acute-rest (median = 90.93) vs acute-stress (median = 122.27), p < 2.22 × 10^−16^; chronic-rest (median = 92.16) vs chronic-stress (median = 105.68), p < 2.22 × 10^−16^, Kolmogorov-Smirnov test). Further, the increase in SPW-R duration was characterized by a rapid onset and sustained increase following stress initiation (Fig. 2D).

Recent work has suggested that longer SPW-Rs could result from the merging of single ripple events, which is reflected as an increase in power in the underlying gamma oscillation (Oliva et al., 2018). To examine this possibility we next measured the low-gamma power (17–40 Hz) during ripple events, but found no differences between stress and rest during both the acute and chronic time points (Fig. 2E and F), suggesting an absence of excessive merging of SPW-Rs. We also examined if stress leads to increased ripple bursts, a phenomenon ascribed to input from the entorhinal cortex (Yamamoto and Tonegawa, 2017). The proportion of ripples occurring as singlets (Fig. 2G) was similar between rest and stress at both the acute and chronic time points (singlets, acute-rest, 0.81 ± 0.02 vs acute-stress, 0.80 ± 0.02, p = 0.999; chronic-rest, 0.78 ± 0.02 vs chronic-stress, 0.78 ± 0.03, p = 1.0, Tukey's HSD) again suggesting that the observed SPW-Rs were longer single events.

We next compared the normalized amplitudes (Supplementary Fig. 2B) of SPW-Rs and found them to be significantly larger during both acute and chronic stress sessions (acute-rest, 4.98 ± 0.21 vs acute-stress, 6.22 ± 0.30, p = 0.009; chronic-rest, 5.14 ± 0.20 vs chronic-stress, 6.50 ± 0.37, p = 0.014, Tukey's HSD). Once again, the increase in SPW-R amplitude showed a rapid onset and sustained increase following the beginning of stress on both the first and last days (Supplementary Fig. 2C). These data demonstrated that stress induction quickly alters basic SPW-R properties in hippocampal area CA1.

### 3.3 Stress alters excitability and participation of CA1 pyramidal cells during SPW-Rs

**Fig. 3:**
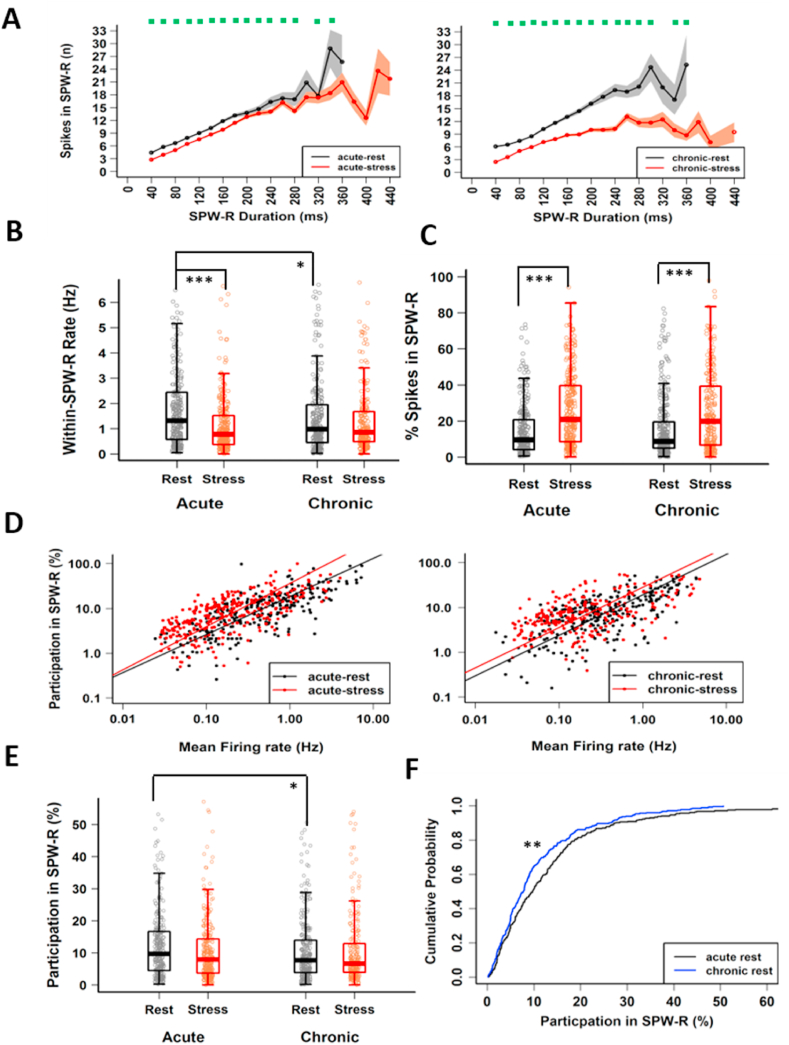
**Altered CA1 pyramidal activation in SPW-Rs during stress-state. (A)** The relationship between SPW-R duration (20-ms bins) and multiunit activity during SPW-Rs for first (left) and last (right) day of CIS (LMMs: behaviour-state, F_(1, 16896)_ = 578.48, p < 2.22 × 10^−16^; duration, F_(16, 16896)_ = 474.38, p < 2.22 × 10^−16^; behaviour-state x duration, F_(16, 16896)_ = 1.955, p = 0.162) and the last day (LMMs: behaviour-state, F_(1, 15877)_ = 1383.89, p < 2.22 × 10^−16^; duration, F_(16, 15877)_ = 358.20, p < 2.22 × 10^−16^; behaviour-state x day, F_(16, 15877)_ = 8.954, p<=0.003) of CIS). Green dots on top of graph indicate significant differences between behaviour-states. **(B)** Within SPW-R average firing rate differs between behaviour-states (two-way ANOVA: behavior-state, F_(1, 1074)_ = 21.262, p = 4.49 × 10^−6^; day, F_(1, 1074)_ = 1.037, p = 0.309; behaviour-state x day, F_(1, 1074)_ = 8.552, p = 0.003; acute-rest vs acute-stress, p = 5.97 × 10^−7^; chronic-rest vs chronic-stress, p = 0.736; acute-rest vs chronic-rest, p = 0.019). **(C)** Percentage of spikes discharged by CA1 pyramidal cells in SPW-R differs between behaviour-states (two-way ANOVA: behaviour-state, F_(1, 1074)_ = 84.83, p < 2.22 × 10^−16^; day F_(1, 1074)_ = 0.079, p = 0.778; behaviour-state x day, F_(1, 1074)_ = 1.35, p = 0.25; Tukey's HSD: acute-rest vs acute-stress, p = 1.4 × 10^−12^; chronic-rest vs chronic-stress, p = 3.62 × 10^−7^). **(D)** Dependency of pyramidal cell participation in SPW-Rs on their mean firing rate on the first day (acute-rest: slope = 0.848, R^2^ = 0.55, p < 2.22 × 10^−16^; acute-stress: slope = 0.96, R^2^ = 0.40, p < 2.22 × 10^−16^; likelihood-ratio test (df = 1) = 4.318, p = 0.038) and the last day (chronic-rest: slope = 0.903, R^2^ = 0.42, p < 2.22 × 10^−16^; chronic-stress: slope = 0.898, R^2^ = 0.21, p = 2.97 × 10^−13^; likelihood-ratio test (df = 1) = 0.006, p = 0.94) of CIS. **(E)** Pyramidal cell participation in SPW-Rs differs with the progression of stress protocol (two-way ANOVA: behaviour-state, F_(1, 1074)_ = 1.614, p = 0.204; day, F_(1, 1074)_ = 4.951, p = 0.026; behaviour-state x day, F_(1, 1074)_ = 1.967, p = 0.161; Tukey's HSD: acute-rest vs acute-stress, p = 0.235; chronic-rest vs chronic-stress, p = 0.999; acute-rest vs chronic-rest, p = 0.043). **(F)** Cumulative distribution of pyramidal cell participation in SPW-Rs differs between rest-states on the first day (black) and last day (blue) of CIS (Kolmogorov-Smirnov test: D = 0.142, p = 0.006). All box-plots, represent interquartile range (IQR, 25th-75th percentiles), median is the thick line in the box and whiskers extend to 1.5 times the IQR. *p < 0.05, **p < 0.01, ***p < 0.001. Acute-rest: n = 288 cells, N = 17 mice; acute-stress: n = 282 cells, N = 16 mice; chronic-rest: n = 282 cells, N = 16 mice; chronic stress: n = 226 cells, N = 16 mice). (For interpretation of the references to colour in this figure legend, the reader is referred to the Web version of this article.)

We further probed for evidence of hyperexcitability during SPW-Rs by comparing the pyramidal cell firing patterns during SPW-R events between rest and stress states during both acute and chronic stress. In agreement with suppressed average firing rates reported above, multiunit activity was muted during SPW-Rs across both stress sessions, though the magnitude of difference was larger during chronic stress (Fig. 3A). While acute stress did not alter the relationship between CA1 pyramidal cell firing in SPW-Rs and their overall mean firing rate (Supplementary Fig. 3A: acute-rest, R^2^ = 0.56, p < 2.22 × 10^−16^; acute-stress, R^2^ = 0.47, p < 2.22 × 10^−16^, likelihood ratio test, p = 0.80) this was not true for chronic stress (chronic-rest, R^2^ = 0.44, p < 2.22 × 10^−16^; chronic-stress, R^2^ = 0.33, p < 2.22 × 10^−16^, likelihood ratio test, p = 0.002). Similar to the change in overall activity, we observed a decrease in pyramidal cell firing rate (Hz) inside SPW-Rs during acute stress (Fig. 3B acute-rest, 2.20 ± 0.19 Hz vs acute-stress, 1.38 ± 0.12 Hz, p = 5.97 × 10^−7^, Tukey's HSD), while following chronic stress, mean firing remained low both during rest and stress on the last day (acute-rest, 2.20 ± 0.19 Hz vs chronic-rest, 1.62 ± 0.11 Hz, p = 0.019, chronic-rest, 1.62 ± 0.11 Hz vs chronic-stress, 1.45 ± 0.12 Hz, p = 0.736, Tukey's HSD). Further, the impact of chronic stress on firing in SPW-Rs during the rest-state was also evident in the cumulative distribution plots (Supplementary Fig. 3B), as the rest-state on the last day showed a leftward shift (acute-rest vs chronic-rest, p = 0.01, Kolmogorov-Smirnov test). Firing outside of SPW-Rs decreased during both the acute-stress and chronic-stress state (Supplementary Fig. 3C). Interestingly, despite this overall drop in activity, during stress-states pyramidal cells discharged a much larger percentage of their total spikes inside of SPW-Rs (Fig. 3C). The fraction of spikes inside SPW-Rs during stress almost doubled on the first day (acute- rest, 14.49 ± 0.85% vs acute-stress, 26.19 ± 1.21% p = 1.4 × 10^−12^, Tukey's HSD), and remained elevated on the last day (chronic-rest, 15.93 ± 1.05% vs chronic-stress, 25.0 ± 1.45%, p = 3.62 × 10^−7^, Tukey's HSD) of CIS.

We next assessed the effects of stress on the participation of pyramidal cells in SPW-Rs events. In agreement with previous reports (Fernández-Ruiz et al., 2019), the baseline firing rate of pyramidal cells displayed a positive relationship with the cell's participation probability in SPW-Rs during both acute and chronic stress, though slight but significant differences were observed during acute stress (Fig. 3D: acute-rest, R^2^ = 0.55, p < 2.22 × 10^−16^; acute-stress, R^2^ = 0.40, p < 2.22 × 10^−16^; likelihood ratio test, p = 0.038; chronic-rest, R^2^ = 0.42, p < 2.22 × 10^−16^; chronic-stress, R^2^ = 0.21, p = 2.97 × 10^−13^, likelihood ratio test, p = 0.94). Further, the extent of pyramidal cell participation in SPW-Rs varied widely (Grosmark et al., 2012; Ylinen et al., 1995) and a comparison across sessions and days revealed a significant effect of day (Fig. 3E). While pyramidal cell participation was similar on the first day (acute-rest, 13.39 ± 0.84% vs acute-stress, 11.41 ± 0.73%, p = 0.235, Tukey's HSD), the chronic rest-state showed a significantly lower participation than that of the acute rest-state (acute-rest, 13.39 ± 0.84% vs chronic-rest, 10.57 ± 0.58%, p = 0.043, Tukey's HSD), suggesting that chronic stress suppresses pyramidal cell participation in SPW-Rs. No further decrease was observed between behaviour states on the last day (chronic-rest, 10.57 ± 0.60% vs chronic-stress, 10.77 ± 0.73%, p = 0.999, Tukey's HSD) of CIS. Cumulative distribution plots further confirmed the impact of chronic stress on SPW-R participation during the rest state (Fig. 3F: acute-rest vs chronic-rest, p = 0.006, Kolmogorov-Smirnov test). The suppressed cell participation on day 10 was not caused by our ripple detection approach, as we confirmed suppressed participation in response to stress even when we used fixed length SPW-Rs (±200 ms from the peak of SPW-Rs), as well as after adjusting the 3-sd threshold in LFP ripple power to include smaller (lowering it to 2-sd) or larger events (increasing it to 4-sd) respectively (Supplementary Fig. 3D). Further, narrowing our filter settings for SPW-R detection to 120–250 Hz also produced similar results (Supplementary Fig. 3E).

Previous studies have reported that deep and superficial CA1 pyramidal neurons display different firing and bursting profiles (Mizuseki et al., 2011) and also differ in their association with SPW-Rs (Valero et al., 2015), raising the possibility that phenotypes described here could be confounded by having a greater proportion of superficial pyramidal cells on the last day of CIS. While tetrode recordings do not allow us to address this question directly, a greater fraction of superficial cells should lead to lower bursting activity. Thus, we next calculated burst index (spikes in burst/total spikes) and found no significant difference between rest-states across days (acute-rest, 0.375 ± 0.009 vs chronic rest, 0.384 ± 0.01; Tukey's HSD = 0.983). The absence of any significant differences in burst index or inter-burst-interval suggests that the effects of stress on day 10 cannot be explained by differences in the proportions of deep and superficial CA1 pyramidal cells. Furthermore, to rule out the possibility that suppressed cell participation on the last day was caused by a deterioration in the quality of the electrophysiological recordings, we calculated isolation distance, a cluster quality parameter that estimates how distant the clustered spikes are from other spikes recorded on the same electrodes. No significant differences in isolation distance was found between rest-states on day first and last (acute-rest, 32.01 ± 1.34 vs chronic rest, 33.16 ± 1.13, Tukey's HSD = 0.245).

Overall, these data demonstrated that despite suppressed firing rates, SPW-R-specific activation of CA1 pyramidal cells was enhanced during both acute and chronic stress. However, with progression of the CIS protocol, pyramidal cells participated in fewer SPW-Rs during the rest period adjacent to the stress exposure.

### 3.4 Co-firing of CA1 pyramidal cells is altered by stress

**Fig. 4:**
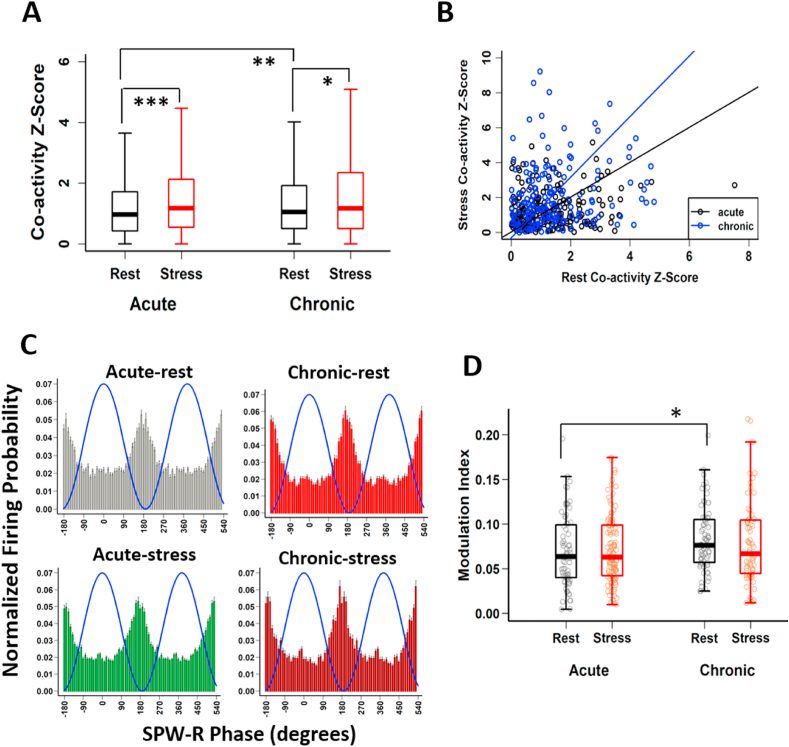
**Altered CA1 pyramidal cell coactivation in SPW-Rs during stress-state. (A)** Co-activity Z-scores of pyramidal cells during SPW-Rs differ between rest-state (black) and stress-state (red) (two-way ANOVA: behaviour-state, F_(1, 6195)_ = 41.286, p = 1.41 × 10^−10^; day, F_(1, 6195)_ = 4.676, p = 0.031; behaviour-state x day, F_(1, 6195)_ = 4.861, p = 0.027; Tukey's HSD: acute-rest, n = 1625 cell pairs vs acute-stress, n = 1610 cell pairs, p = 4.04 × 10^−9^; chronic-rest, 1701 cell pairs vs chronic-stress, n = 1263 cell pairs, p = 0.025; acute-rest vs chronic-rest, p = 0.0035). **(B)** Relationship between cell pairs of positive co-activity Z-scores during both rest-state and stress-state significantly differs between days (acute: slope = 0.99, R^2^ = 0.022, p = 0.033 n = 208 cell pairs; chronic: slope = 1.74, R^2^ = 0.019, p = 0.076, n = 165 cell pairs; likelihood-ratio test, p = 1.28 × 10^−7^) of CIS. **(C)** Group histograms for pyramidal cell spiking during SPW-Rs for acute-rest (grey), acute-stress (green), chronic-rest (red) and chronic-stress (maroon). The Blue lines on each subpanel represents the phase of the SPW-R. **(D)** Modulation index differs slightly between days (LMMs: behaviour-state, F(1, 311) = 0.016, p = 0.900; day, F(1,311) = 6.335, p = 0.012; behaviour-state x duration, F(1, 311) = 1.294, p = 0.256; Tukey's HSD: acute rest vs chronic rest, p = 0.049). Boxplots represent interquartile range (IQR, 25th-75th percentiles), median is the thick line in the box and whiskers extend to 1.5 times the IQR. *p < 0.01, **p < 0.005, ***p < 0.001. Acute-rest: N = 17 mice; acute-stress, N = 16 mice; chronic-rest, N = 16 mice; chronic-stress, N = 16 mice). (For interpretation of the references to colour in this figure legend, the reader is referred to the Web version of this article.)

Hippocampal neuronal synchrony peaks during SPW-Rs (Buzsáki et al., 1992; Csicsvari et al., 1999b; Wilson and McNaughton, 1994) and this has been suggested as a key mechanism in hippocampal mnemonic function (Buzsáki and Mizuseki, 2014; Cheng and Frank, 2008). Our finding that a greater fraction of pyramidal spiking occurs inside SPW-Rs, raised the possibility of enhanced synchrony of pyramidal cell firing during stress. However, the overall decrease in firing rates, and reduced SPW-R participation by pyramidal cells with repeated stress, suggested the contrary. To examine this, we quantified the co-activation of pairs of pyramidal cells exhibiting a positive correlation in their firing patterns in a given session and observed a significant elevation during stress periods (Fig. 4A). Co-activity Z-scores were significantly higher during acute (acute-rest, 1.228 ± 0.027 vs acute-stress, 1.495 ± 0.031, p = 4.04 × 10^−9^, Tukey's HSD) as well as chronic stress (chronic-rest, 1.388 ± 0.03, vs chronic-stress, 1.622 ± 0.043, p = 0.025, Tukey's HSD), compared to rest states. Further, the rest-state on the last day showed significantly larger co-activation values as compared to the first day (acute-rest vs chronic-rest, p = 0.0035, Tukey's HSD). This enhanced co-activity was specific to SPW-R events, as outside of SPW-Rs an overall significant decrease was observed in both stress sessions (acute-rest, 4.651 ± 0.039 vs acute-stress, 4.187 ± 0.043, p < 2.22 × 10^−16^, Tukey's HSD; chronic-rest, 4.629 ± 0.04, vs chronic-stress, 4.110 ± 0.048, p < 2.22 × 10^−16^, Tukey's HSD), with no difference between rest-states (acute-rest vs chronic-rest, p = 0.982, Tukey's HSD).

Next, to compare the coordinated activity more directly between states, we calculated co-activity scores of cell-pairs with positive values recorded during both rest and stress states (Fig. 4B). This analysis revealed a positive relationship between cell pairs during both rest and stress states on the first day (acute: R^2^ = 0.022, p = 0.033), which grew stronger with further episodes of stress over time (chronic: R^2^ = 0.019, p = 0.076) and significantly differed between days (p = 1.28 × 10^−7^, likelihood ratio test), suggesting that chronic stress led to reorganization of network activity even during non-stress periods.

Finally, we asked if the stress-associated increase in synchrony was related to changes in the phase-locking of CA1 pyramidal cells to the SPW-R oscillation. We found that the fraction of pyramidal cells phase-locked to SPW-Rs was significantly higher during stress-state on day 1 (acute rest: 72/156 (46%); acute stress: 106/167 (63%), p = 0.003, chi-square test), but not on day 10 (chronic rest: 73/144 (51%); chronic stress: 64/106(48%), p = 0.164, chi-square test). In agreement with previous studies (Csicsvari et al., 1999b; Witton et al., 2016; Ylinen et al., 1995), in all sessions, pyramidal cells displayed preferred spiking during the trough of the SPW-Rs (Fig. 4C). The mean phase preference did not differ between behaviour states (circular ANOVA = F_(3, 311)_ = 2.073, p = 0.104) and the modulation index (MI) that measures average strength of phase modulation (Gu et al., 2013) was similar in acute-rest and acute-stress. However, MI did show a marginal increase during the rest-state (Fig. 4D) on day 10 compared to rest-state on day 1 (acute-rest vs chronic-rest, p = 0.049, Tukey's HSD).

## 4 Discussion

Accumulating evidence has identified stress-induced changes in the hippocampus across biological scales – from behavioural deficits to their neuronal, synaptic and molecular correlates. However, how the intact hippocampal circuitry in an awake behaving animal responds to, and encodes information during a stressful situation is poorly understood (McEwen, 2007). Here, we report a reduction in spiking in CA1 pyramidal neurons during the first (acute) and last (chronic) exposure to 2-h sessions of immobilization stress (Fig. 1D and E). During both acute and chronic stress, theta/delta ratio remained low (Fig. 1G) and SPW-R events were of longer duration compared to the rest (Fig. 2B and C). Further, stress altered the firing of CA1 pyramidal cells during SPW-Rs, leading to a greater fraction of spikes occurring inside SPW-Rs during both acute and chronic stress (Fig. 3C). While there were no changes in the timing or strength of modulation of spiking relative to SPW-R oscillations during stress (Fig. 4C and D), we did observe event specific increases in co-activity, which was also carried over to the rest-state on the final day of chronic stress (Fig. 4A and B).

Considering that stress leads to elevated levels of glutamate and calcium, a long-held view in research on stress-induced plasticity is that stress leads to enhanced excitability in hippocampal circuits (Gunn and Baram, 2017; Sapolsky et al., 1986). However, our *in vivo* recording data revealed a robust decrease in firing rates of CA1 pyramidal cells during acute stress (Fig. 1D). Despite differences with *in vitro* studies, this result is consistent with a previous *in vivo* analysis that found suppression of pyramidal ‘place cell’ activity in an immobilized rat even when it was moved through its place field on a track (Foster et al., 1989), as well as other studies which reported suppressed place cell firing rates after the termination of stress (Park et al., 2015; Passecker et al., 2011). When we measured hippocampal activity by assessing spiking patterns of pyramidal cells during SPW-Rs, which are referred to as “built in” sensors of hippocampal excitability (Buzsáki, 2015), we observed a striking increase in the fraction of spikes that pyramidal cells discharged inside SPW-Rs during stress states (Fig. 3C). This analysis thus revealed evidence in support of event-specific stress-induced enhanced excitability.

Our analyses also revealed stress-induced enhancement in CA1 neuronal synchrony as evidenced by increased pyramidal cell co-activation during SPW-Rs (Fig. 4). Such co-activity during repeated exposures to stress creates conditions at the synaptic level that are consistent with the synaptic saturation hypothesis of stress (Diamond et al., 2004). This gives rise to questions that will require further investigation, including if such synchronous activity would saturate synaptic mechanisms that occlude further synaptic plasticity necessary for subsequent encoding. If so, this may offer insights into earlier findings that chronic stress causes rigidity in hippocampal networks (Tomar et al., 2015), thus leading to context generalization (Krugers H.J. et al., 1997) and spatial learning deficits (Kim et al., 2007; Park et al., 2015). In light of a recent study that links SPW-R duration to learning (Fernández-Ruiz et al., 2019), our findings of increased pyramidal cell synchrony, along with long-duration SPW-Rs suggests that during stress, the hippocampus may encode aversive memories related to the stressful experience (Cadle and Zoladz, 2015; Diamond et al, 2004, 2007; Schwabe et al., 2012).

The observation that altered SPW-R properties were present from the onset of stress (Fig. 2D and Supplementary Fig. 2C) also raises interesting questions about underlying mechanisms. For example, this timeline suggests that both altered SPW-R characteristics and suppressed hippocampal spiking during acute stress are mediated by sympatho-adrenal medullary (SAM) system-activated neurochemicals (Cadle and Zoladz, 2015; Gunn and Baram, 2017). Further, reports that norepinephrine application in hippocampal slices alters SPW-R properties (Haq et al., 2012) and suppresses CA1 pyramidal spiking (Bergles et al., 1996; Pang and Rose, 1987), suggests norepinephrine release as a possible mechanism contributing to the stress phenotypes observed in this study.

During SPW-R events inhibitory neurons display sustained spiking (Csicsvari et al., 1999a; Klausberger et al., 2003; Varga et al., 2012), while CA1 pyramidal cells are only able to fire during narrow windows of reduced inhibition (English et al., 2014; Malerba et al., 2016; Stark et al., 2014), increasing temporal correlations of firing during SPW-Rs (see review by Buzsáki, 2015). Knowing that norepinephrine application tilts CA1 inhibitory-excitatory (I/E) balance in favor of inhibition (Pang and Rose, 1987), it is likely that the stress-induced rapid release of norepinephrine may contribute to enhanced co-activation inside SPW-Rs, particularly on the first day (acute-stress). However, on the last day (i.e., chronic-rest and chronic-stress), additional factors, including CA1 dendritic debranching, elevated basal corticosterone levels and altered CA3-CA1 connectivity could also come into play. Finally, our findings of suppressed CA1 spiking during the chronic states is in agreement with the role of glucocorticoid receptor (GR)-mediated suppression of hippocampal neuronal activity during stress (Joëls, 2018).

Following repeated stress, the enhanced pyramidal cell co-activation during SPW-Rs was also accompanied by participation of CA1 pyramidal cells in fewer SPW-Rs than during non-stress states (Fig. 3E and F). Whether suppressed participation of pyramidal cells is an epiphenomenon or counters enhanced network synchrony as stress becomes chronic is not clear. However, these results show that repeated stress alters hippocampal ripple-spike interactions, a phenomenon previously linked to altered cognition in rodent models of disease (Middleton et al., 2018; Raveau et al., 2018; Suh et al., 2013; Witton et al., 2016).

In the absence of EMG data in this study, we cannot differentiate between SPW-Rs which occur during slow-wave sleep and those that occur during quiet wakefulness; thus, future studies are needed to assess if stress differentially impacts these events. This was designed as a within-subject longitudinal study, and consequently, we did not have a control group, thus raising the question of whether altered ripple-spike-interactions were caused by stress or other extraneous factors such as familiarity/novelty (Frank et al., 2004; Karlsson and Frank, 2008) or habituation (Grégoire et al., 2014; Longordo et al., 2011; Vecsey et al., 2013). We believe such extraneous factors had minimal impact on the changes elicited by stress described here for several reasons. First, the mice were very familiar with the conditions (see Methods). Second, novelty is associated with increased firing of pyramidal cells, but during acute stress we observed suppressed firing. Third, during both acute-rest and chronic-rest, the experimental conditions were identical, i.e., mice rested in a small, high-walled, opaque, sleep-box, with minimal opportunity to explore. Thus, behaviourally, the rest-states were very similar to stress-states, ruling out immobility as a major contributing factor. Finally, decreased firing and participation of pyramidal cells, along with increased co-activity of cell-pairs inside SPW-Rs were observed during acute-stress and chronic-rest, suggesting altered hippocampal ripple-spike-interactions were not behaviour-specific and cannot be explained by broad time-scale extraneous factors such as familiarity, habituation, and immobility, but rather are driven by stress-induced processes operating on a much faster timescale.

Two major afferents to area CA1 that influence information processing and SPW-R properties are the Schaffer collaterals from area CA3 (Davoudi and Foster, 2019; Nakashiba et al., 2009) and temporoammonic inputs from the entorhinal cortex (Oliva et al., 2018; Yamamoto and Tonegawa, 2017). We found no change in either SPW-R-associated low-gamma power (Fig. 2F) or ripple-burst patterns (Fig. 2G), suggesting that the stress phenotypes we observed are likely caused by changes occurring within areas CA1 and CA3 and are minimally influenced by entorhinal inputs. In addition, extrahippocampal regions, including the amygdala, are known to influence CA1 neural dynamics (Ghosh et al., 2013; Kim et al, 2012, 2015), LTP (Kim et al., 2005; Vouimba and Richter-Levin, 2005) and the release of stress hormones, including catecholamines (Roozendaal et al., 2009; Schwabe et al., 2012). Thus, future studies will be needed to characterize the relative contributions of CA3 and amygdalar inputs to SPW-R duration, pyramidal cell synchrony, and altered participation of pyramidal cells in SPW-Rs, reported in the current study.

In conclusion, a large body of earlier work has characterized the morphological, molecular, physiological, and behavioural changes in the hippocampus following either acute or repeated stress. Our study adds a new layer of understanding by providing a window into the dynamics of hippocampal network activity during episodes of stress and identifies altered ripple-spike interactions as a potential biomarker of stress.

## Correspondence and request for material

Any request for data or code should be addressed to A.T. or T.J.M.

## CRediT authorship contribution statement

**Anupratap Tomar:** Conceptualization, Investigation, Formal analysis, Data curation, Writing – original draft, Writing – review & editing, Visualization. **Denis Polygalov:** Software, Formal analysis, Data curation, Writing – review & editing. **Sumantra Chattarji:** Conceptualization, Writing – original draft, Writing – review & editing. **Thomas J. McHugh:** Conceptualization, Formal analysis, Writing – original draft, Writing – review & editing, Project administration, Funding acquisition.

## Declaration of competing interest

The authors declare no competing financial interests.
