# Supplementary material for: Stress enhances hippocampal neuronal synchrony and alters ripple-spike interaction
Source: Neurobiol Stress. 2021 Apr 13;14:100327. doi: 10.1016/j.ynstr.2021.100327 (PMC8079661; doi:10.1016/j.ynstr.2021.100327)
Supplement: Multimedia component 1 [file mmc1.docx]

**Appendix**

**Stress enhances hippocampal neuronal synchrony and alters ripple-spike interactions**

**Supplementary figures**


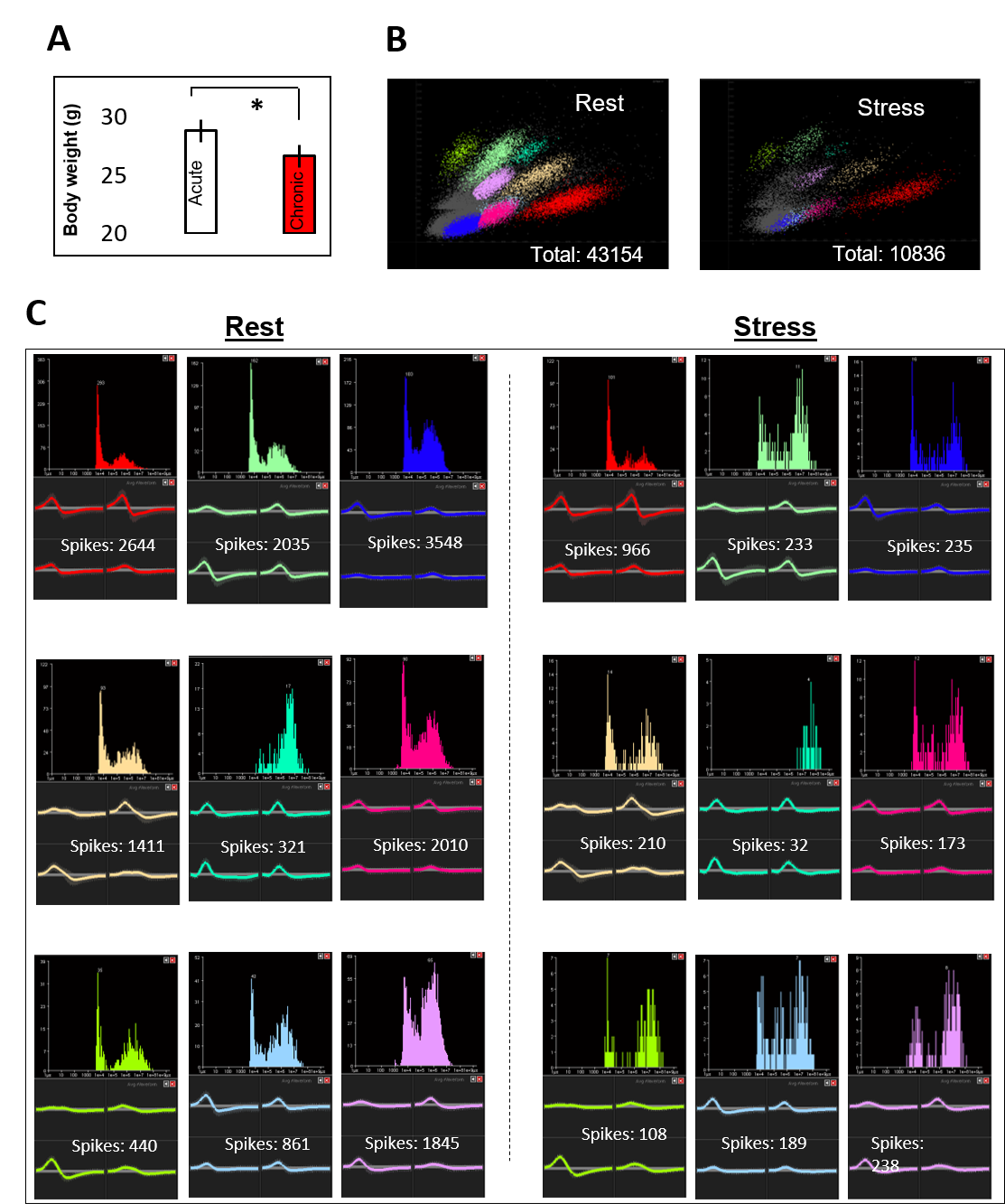


**Supplementary Fig. 1. Chronic stress alters body weight and firing rate of CA1 pyramidal neurons (A)** Change in absolute body weight with the progression of CIS protocol (acute, 28.82 ± 0.97 vs chronic, 26.65 ± 0.69, N = 4 mice, paired t-test: t = 3.8411, p = 0.031). **(B)** Time matched (30 min) representative examples of unsorted spikes recorded during rest-state (left) and stress-state (right). Numbers next to each cluster depict total number of spikes recorded during that state. **(C)** Each subpanel during rest-state (left) and stress-state (right) contains inter-spike-intervals (top) and waveforms (bottom) on each wire of a tetrode from all 9 cells sorted in b. Numbers on each subpanel represents total number of spikes discharged by that specific cell. * p < 0.05.

**
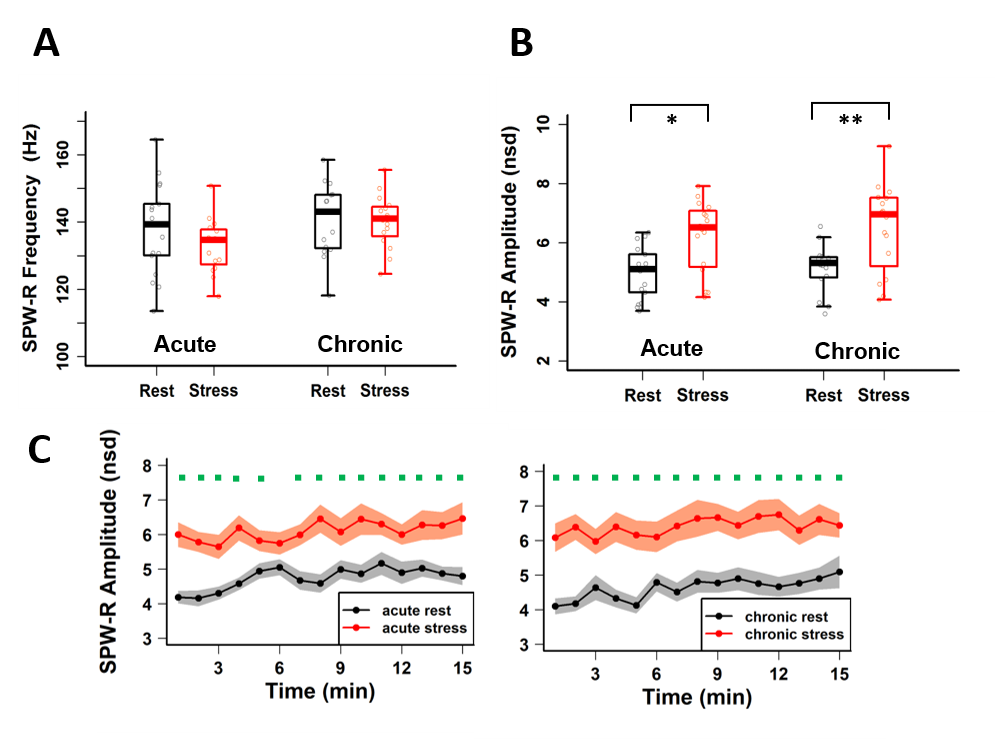
Supplementary Fig. 2. Effects of repeated stress on SPW-R properties (A)** Frequency of SPW-R oscillations did not differ between rest-state and stress-state (two-way ANOVA: behavior-state, F_(1 ,61)_ = 1.466, p = 0.231; day, F_(1, 61)_ = 4.806, p = 0.032; behavior-state x day, F_(1, 61)_ = 0.801, p = 0.37). **(B)** SPW-R amplitude differs between rest-state and stress-state (two-way ANOVA: behavior-state, F_(1 ,61)_ = 20.607, p = 2.7110^-5^; day, F_(1, 61)_ = 0.598, p = 0.442; behavior-state x day, F_(1, 61)_ = 0.005, p = 0.945; Tukey’s test: acute-rest vs acute-stress, p = 0.009; chronic-rest vs chronic-stress, p = 0.014). **(C)** Temporal dynamics of averaged SPW-R amplitudes (1-min bins) differ between behavior states on first day (left: LMMs: behavior-state, F_(1, 447)_ = 252.08 p < 2.22x10^-16^; minutes, F_(14, 447)_ = 1.95, p = 0.021; behavior-state x minutes, F_(14, 447)_ = 1.11, p = 0.35) and the last day (right: LMMs: behavior-state, F_(1, 412)_ = 288.20, p < 2.22x10^-16^; minutes, F_(14, 412)_ = 2.447, p = 0.003; behavior-state x minutes, F_(14, 412)_ = 1.212, p = 0.26) of CIS. Green dots on the top of graph indicate significant differences between behavior-states. nsd represents unit of ripple amplitude in number of standard deviations. The boxes in the box plots, represent interquartile range (IQR, 25^th^-75^th^ percentiles), median is the thick line in the box and whiskers extend to 1.5 times the IQR. * p < 0.05, ** p < 0.01, *** p < 0.001. Acute-rest, N = 17 mice; acute-stress, N = 16 mice; chronic stress, N = 16 mice; chronic stress, N = 16 mice).


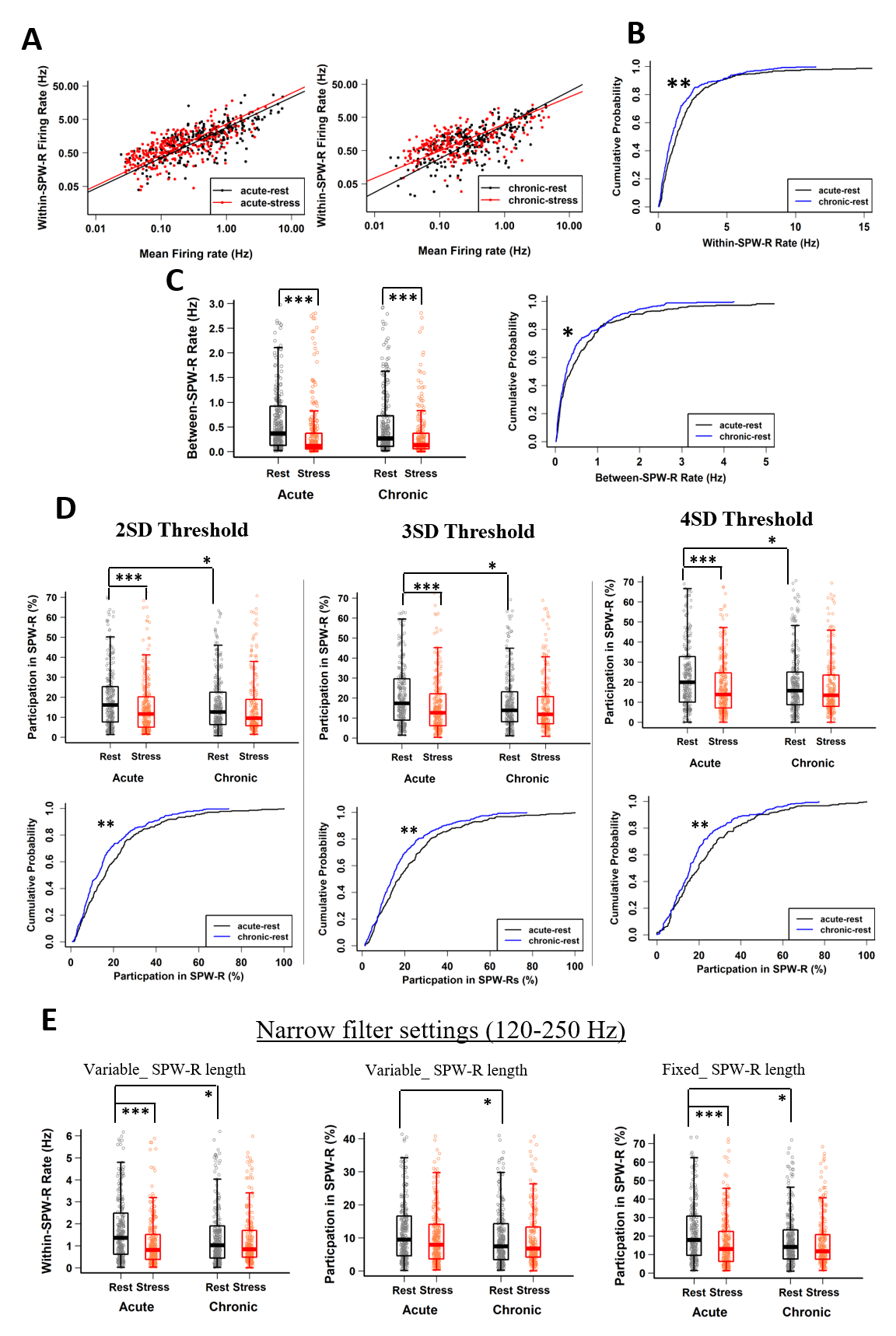


**Supplementary Fig. 3. Stress decreases pyramidal cell spiking and participation in SPW-Rs (A)** Dependency of CA1 pyramidal cell firing rate in SPW-Rs on their mean firing rate was not affected by either acute (acute-rest: slope = 0.907, R^2^ = 0.56, p < 2.22x10^-16^; acute-stress: slope = 0.92, R^2^ = 0.47, p < 2.22x 10^-16^; likelihood-ratio test (df = 1) = 0.063, p = 0.80) or chronic stress (chronic-rest: slope = 1.011, R^2^ = 0.44, p < 2.22x10^-16^; chronic-stress: slope = 0.81, R^2^ = 0.33, p < 2.22x10^-16^; likelihood-ratio test (df = 1) = 9.40, p = 0.002). **(B)** Cumulative distribution plots for within SPW-R firing rate differ between rest-states on the first (black line) and the last (blue line) day of CIS (acute-rest vs chronic-rest: Kolmogorov-Smirnov test, D = 0.138, p = 0.01). **(C)** Between SPW-R average firing rate differs (left) between behavior states (two-way ANOVA: behavior-state, F_(1,1074)_ = 85.385, p < 2.22 x10^-16^; day, F_(1,1074)_ = 0.272, p = 0.602; behavior-state x day, F_(1,1074)_ = 5.048, p = 0.025; Tukey’s HSD: acute-rest vs acute-stress, p = 6.48 x10^-13^; acute-rest, vs chronic-rest, p = 0.110; chronic-rest vs chronic-stress, p = 1.88x10^-5^). Cumulative distribution plots (right) of between SPW-R firing rate differ between rest-states on the first (black line) and the last (blue line) day of CIS (acute-rest vs chronic-rest: Kolmogorov-Smirnov test, D = 0.119, p = 0.036). **(D)** Pyramidal cell participation during SPW-Rs was suppressed on day 10 and remained unaffected by fixing the ripple length or modifying the detection threshold of SPW-Rs to 2SD, 3SD or 4SD. **(E)** Pyramidal cell firing rate (left) and participation (middle, right) remained significantly reduced on day 10 when a narrower filter band (120-250 Hz) was used for SPW-R detection. The boxes in the box plots, represent interquartile range (IQR, 25^th^-75^th^ percentiles), median is the thick line in the box and whiskers extend to 1.5 times the IQR. * p < 0.05, ** p < 0.01. Acute-rest: n = 288 cells, N = 17 mice; acute-stress: n = 282 cells, N = 16 mice; chronic-stress: n = 282 cells, N = 16 mice; chronic-stress: n = 226 cells, N = 16 mice.
